# Theory-based predictors of prescribing behaviour for neurodegenerative diseases: A cross-sectional survey with European healthcare professionals

**DOI:** 10.1371/journal.pone.0353479

**Published:** 2026-07-15

**Authors:** Emma Begley, Jason Michael Thomas, William Hind, Carl Senior

**Affiliations:** 1 School of Psychology, College of Health and Life Sciences, Aston University, Birmingham, United Kingdom; 2 Alpharmaxim, Century Park, Altrincham, United Kingdom; Isra University Faculty of Pharmacy, JORDAN

## Abstract

**Objectives:**

Explore the likelihood of healthcare professionals (HCPs) prescribing a new, novel or different to usual medication to patients with a newly diagnosed early onset or advanced stage neurodegenerative disease (ND), and identification of the theoretical behavioural determinants that underpin these predictors.

**Design:**

A quantitative cross-sectional study using a newly developed survey underpinned by the Theoretical Domains Framework (TDF) was delivered online between September 2023 and March 2024.

**Setting:**

All levels of healthcare where medication is prescribed for NDs across Austria, France, Germany, Italy, the Netherlands, Spain, Sweden and the UK.

**Participants:**

Recruited predominately via a participation panel, HCPs who have prescribed medication for a ND in the past 2 years were eligible to take part, with 279 participants (66% male) completing the study in full.

**Primary and secondary outcome measures:**

The primary outcome was HCPs’ likelihood of prescribing a new, novel or different to usual medication compared with their routine choice for patients with a newly diagnosed early onset or advanced stage ND. A secondary outcome was to identify factors aligned to the TDF that influenced prescribing and describe them using the Capability, Opportunity and Motivation – Behaviour (COM-B) model.

**Results:**

HCPs were significantly more likely to consider prescribing outside of routine habit for patients with advanced stage ND compared to patients with new early onset diagnosis (60.8% [95% confidence interval (CI) 57.70, 63.95] *vs* 51.8% [95% CI 48.61, 55.05], respectively). Logistic regression models revealed a total of four factors that predicted prescribing. In the scenario with newly diagnosed patients, HCP intention (β = 0.324, 95% CI 4.75, 10.69), behavioural regulation (β = 0.191, 95% CI 1.57, 8.92), and social/professional role and identity (β = ‑0.184, 95% CI 1.57, 8.92) were each significant predictors of prescribing. In the scenario with patients with advanced stage ND, HCP intention also predicted prescribing (β *=* 0.234, 95% CI 31.02, 65.15), along with HCP belief about consequences (β = −0.145, 95% CI 2.73, 8.11). Thus, the TDF determinants that predicted prescribing indicate that COM-B capability (TDF behavioural regulation) and motivation (TDF intention, social/professional role and identity, and belief about consequences) domains may be relevant targets for changing prescribing habits.

**Conclusions:**

There is a complex interplay of specific and generic predictors that influence prescribing for patients with a newly diagnosed early onset or advanced stage ND. Appropriate behaviour change frameworks that target HCPs’ capability and motivation should be considered to support changes towards the optimal use of new, novel or different to usual medication in the future.

## Introduction

Neurodegenerative diseases (NDs), such as Parkinson’s disease (PD), are a leading cause of disability and death worldwide [1]. Year on year, the incidence and prevalence rates for NDs have increased, and there is growing concern that the burden of these diseases will double across the globe by 2043 [2–4]. In the absence of a cure, various medications are used to manage the symptoms of NDs [5]; however, the variability of patient symptoms, health priorities and drug effectiveness are challenging obstacles that healthcare professionals (HCPs) often face when determining the most appropriate medication to prescribe [6,7]. Hence, there is a need to understand the role of the contextual influences at play when HCPs make medication choices, so that efforts to optimise prescribing can be established.

Much is already understood about factors that influence prescribing choices. For example, a recent systematic review identified patient age and type of prescriber (e.g., general practitioner or neurologist) as key determinants for prescribing medication to patients with PD [8]. More broadly, a qualitative study reported that factors such as the severity of patients’ health concerns, physicians’ preferred approaches, and availability of resources influenced clinical decision making for patients with complex multimorbidity [9]. The psychology of decision making has also been explored, with some evidence indicating that various heuristic strategies lead to cognitive biases (e.g., availability or outcome bias) that influence HCP judgement [10]. The factors reported in these studies add valuable knowledge about prescribing; however, little is known about which factors are most important or how they can be used to optimise prescribing behaviour.

Enhancing treatment choice requires an in-depth understanding of prescribing behaviour to identify what needs to change and how to effectively bring about the desired change. The behaviour change wheel (BCW) is a leading theoretical framework that guides users through the identification of barriers and determinants that underpin an individual’s Capability, Opportunity and Motivation to perform a Behaviour (COM-B) [11]. This enables users to subsequently identify the most effective approaches (e.g., education or training) and behaviour change technique (BCT; e.g., goal setting or use of a creditable source) to change the targeted determinant(s) [12]. The assessment of behavioural determinants can also be explored by using the Theoretical Domains Framework (TDF), which comprises 14 behavioural determinants that map onto behaviour change components of the COM-B model [13]. The advantage of using both the TDF and COM-B model together is that they provide a greater level of understanding of the theoretical constructs underpinning a behaviour. For example, a review investigating interventions that optimise prescribing reported that 1) knowledge and 2) memory, attention and decision making were two of the most common TDF behavioural determinants identified [14]. Identifying these TDF determinants allowed the authors of the review to use the BCW manual and its associated BCT taxonomy to retrospectively identify which BCTs (e.g., prompts and defaults) could be used to influence a change in behaviour.

There is a clear research gap to understand more about the decision-making drivers behind prescribing. This is a fundamental element of medical practice, and an area where HCPs report difficulty and discomfort when making clinical decisions [15,16]. Exploring and better understanding the factors that influence prescribing for patients with NDs offers an opportunity to improve the prescribing pathway by supporting HCPs to optimise their treatment choices. Pharmacotherapy is the dominant treatment regimen for NDs [17]. For patients with PD specifically, the use of the dopaminergic precursor levodopa as first-line monotherapy is most prescribed [18]. Some studies have indicated that levodopa therapy is prescribed in up to 70% of cases in the USA [19]. The treatment of Alzheimer’s disease is not dominated to the same degree as that of PD but cholinesterase inhibitors, such as donepezil, still account for 60% of the first-line treatments in the USA [20].

This study aimed to identify the factors that predict HCP prescribing decisions in order to demonstrate the potential to optimise treatment choice. It is hypothesised that the factors which influence prescribing choices, and the behavioural determinants that they map to, will vary depending on the clinical presentation of a patient.

## Methods

### Study design

A quantitative cross-sectional study, using online survey data, was conducted between September 2023 and March 2024 (see supplementary data S1 File for the STROBE checklist). The survey was designed to be completed online to facilitate wider data collection from participants eligible to take part across Europe. The primary outcomes were scores derived from participant data that indicated how often they are likely to prescribe a new (e.g., a new drug within an established class of drugs), novel (e.g., a newly developed drug class) or different to usual medication (e.g., non–first-line medication) based on two situational prescribing scenarios: scenario 1) patients with newly diagnosed ND and scenario 2) patients with advanced stage ND. Fourteen predictors, each reflecting one of the 14 TDF behavioural determinants [13], were investigated as potential drivers of behaviour. Each predictor was measured using a set of discrete single-item questions that captured data on a 7-point Likert scale (ranging from strongly disagree to strongly agree).

### Participants and recruitment

Participants were HCPs, defined here as any individual who is employed by a healthcare organisation that medically manages or treats patients, including consultants, specialist nurses, general practitioners and pharmacists. To be eligible, participants needed to be a current practicing HCP who has prescribed medication to patients with a ND within 2 years of the data collection period. Participants were excluded if they lived or worked outside of Europe or if they took part in a previous related study [21]; this reduced the risk of participant bias due to exposure to information that informed the development of this study.

Participants were recruited using a two-stage strategy. Firstly, a direct approach was employed where a) prospective participants were identified from relevant conferences or websites and emailed an invitation and b) recruitment posters were shared via social media sites and through relevant ND societies. Participants recruited via this first approach were given the choice of opting into a charity prize draw, whereby the research team identified winners using a random number generator and donated a first prize of £/€100 and two second prizes of £/€50 to a charity of the participant’s choice. Secondly, Qualtrics panel management [22] was used to enable access to recruit a proportionate sample of HCPs from five countries in Europe on behalf of the research team.

In all, 415 participants responded to the invitation to take part and, of these, 393 completed eligibility data. Participants who did not fulfil the eligibility criteria were stopped from continuing with the online survey. This resulted in 362 eligible participants; however, 83 responses were excluded due to incomplete or uninterpretable responses. The final sample, where all data were available for analysis, comprised 279 participants. All participants were provided with a participant information sheet, informed that their participation was voluntary and they could withdraw at any point, and electronically provided their consent by checking boxes via the online survey. Ethical approval for the study was granted from Aston University College of Health and Life Sciences Research Ethics Committee (REF HLS21064).

### Sample size

G*Power version 3.1.9.7 was used to establish the minimum sample size required to predict a small-to-medium effect size (*f*^*2*^ *=* 0.085) to model 14 predictors. Alpha was set at 0.05 and power at 80%, which indicated that a minimum sample size of 228 participants was required. To account for incomplete data or data that needed to be excluded (e.g., due to participants providing uninterpretable responses), the recruitment target was set at 250 participants.

### Measures

The online survey comprised three parts: 1) demographic questions, 2) prescribing scenarios to measure the primary outcomes and 3) a long-form 93-item questionnaire to measure the 14 predictors. The survey was presented to respondents in English.

### Demographics

Data were collected regarding participants’ age, gender, country of practice, clinical setting, clinical role, area of ND specialism, years registered as an HCP and rurality. These data provided context to the study population and to test for potential covariates to be used during the data analysis.

### Prescribing scenarios

Two situational prescribing scenarios that describe patients with newly diagnosed and advanced stage ND were used to capture outcome measures that reflect a range of potential real-world prescribing practices (Table 1).

**Table 1:** Prescribing scenarios 1 and 2.

Scenario 1	Scenario 2
You are presented with a patient with newly diagnosed early-onset symptoms of PD/ND who has never received any medication for this disease. The patient reports having fairly good quality of life and is worried about their symptoms impacting their ability to continue working	You are presented with a patient with advanced stage PD/ND who has tried several medications that have not produced a satisfactory therapeutic effect. The patient is retired and reports having a poor quality of life
Reflecting on how you currently prescribe, think about a medication that instinctively comes to mind for this patient. Then, please consider if there are other options that perhaps you have never prescribed before or, for your own reasons, are less of a first choice In practice, on a scale of 0–100 (0 = never and 100 = always), how often do you deviate from what you would instinctively prescribe to prescribing something new, novel or different to usual?	

ND, neurodegenerative disease; PD, Parkinson’s disease

### Long-form 93-item questionnaire

The long-form 93-item questionnaire to measure predictors comprised 14 sets of questions, each of which aligns to one of the 14 domains within the TDF. The number of items within each set varies between 2 and 23; they were developed using a combination of insight generated from a literature review [23] and an HCP consensus study [21] on this topic, with additional items modified from other appropriate published studies [24–31]. Thus, the variation in the number of items per set was due to the range of individual facets that the data insights revealed. For example, within the set of items that aligned with the memory, attention and decision-making domain, 9 items related to patient characteristics and demographics, and a further 10 items were from a validated defensive decision-making questionnaire [27]. For each item aligned with each domain, participants were invited to indicate their response on a 7-point Likert scale ranging from 1 = strongly disagree to 7 = strongly agree (see S2 File Study protocol, Table 1 for each domain from the TDF and examples of corresponding questionnaire content). Reliability of the long-form 93-item questionnaire was assessed using Cronbach’s alpha (α) and enhanced by the removal of individual items from question sets where indicated during the assessment. Eight predictor domains had high reliability (Cronbach’s α ranging between 0.8 and 0.7): emotion, belief about consequences, knowledge, behavioural regulation, skills, reinforcement, social influence and memory, attention and decision making. Six predictor domains had relatively lower reliability (Cronbach’s α ranging between 0.6 and 0.5): belief about capabilities, goals, environmental context and resources, social/professional role and identity, optimism and intentions.

### Procedure

The online survey platform Qualtrics was used to host the survey, enabling participants to complete it by clicking an anonymised URL link using a computer, mobile phone or other device with internet access. After reading a participant information sheet, participants consenting to take part were asked to complete the demographic/eligibility questions. Those who were eligible were prompted to continue and answer the scenario-based questions, followed by the long-form 93-item questionnaire. Those who were not eligible were automatically prevented from continuing any further and thanked for their time. The survey took approximately 20 minutes for participants to complete. A study protocol is available in supplementary data S2 File.

### Data analysis

#### Data processing.

A total of 415 responses were downloaded from Qualtrics and processed in Microsoft Excel. After removing any ineligible (n = 53), incomplete (n = 27) or uninterpretable (n = 56) responses/data, 279 complete responses were imported to IBM SPSS Statistics 29 for statistical analysis. For the long-form 93-item questionnaire, individual item scores within each domain of the TDF were added together and averaged (e.g., the scores to the eight knowledge questions within the knowledge domain were added together and averaged) so that a single independent variable was created to represent each of the 14 domains. Where response scales to individual items were in different directions within a domain (e.g., strongly agreeing with one item might indicate something positive, but strongly agreeing with another item might indicate something negative), item responses were inverted (as appropriate) so that all responses were aligned in the same direction prior to averaging.

#### Covariates.

Pearson’s correlation coefficient was used to establish whether any of the demographic variables correlated with either of the two outcome dependent variables (i.e., scenarios 1 and 2). IBM SPSS Statistics 29 was used for all statistical analysis. Where correlations were statistically significant (*p* < 0.05) and the *r* value was equal to or exceeded 0.2, the covariate would be entered into the regression.

#### Main analyses.

A multiple stepwise linear regression model was created. Fourteen predictor variables, each representing the domains of the TDF (as described above), were entered into the model. The model was run once to predict the outcome scores derived from scenario 1 (patient with newly diagnosed ND) and then again to predict the outcome scores derived from scenario 2 (patient with advanced stage ND), in both instances, to determine the predictors of how often participants would prescribe a new, novel or different to usual medication. Multicollinearity was investigated and, where identified, items were averaged across or removed.

#### Patient and public involvement.

No patients were involved in developing the design, data collection, analysis or write up of this study. However, to establish the acceptability and suitability of the survey, feedback on the final version was gathered from four HCPs who took part in a previous linked study [21]. HCP comments were positive and there were no amendments suggested.

## Results

### Participant characteristics

Data were analysed for 279 HCPs. Males represented most of the sample (n = 184, 65.9%) and, overall, participants had a mean of 41.93 years of age (standard deviation [SD] 8.19). Participants were registered practitioners for an average of 11.70 years (SD 6.72), with the highest proportion of participants reporting their role as ‘medical doctor’ (n = 135, 48.4%). The majority of participants were recruited from the UK, Italy, Germany, France and Spain (97.8%) and the following characteristics were reported by a majority of participants: practicing in a primary care setting (n = 155, 55.6%), specialising in PD (n = 241, 86.4%) and working in an urban locality (n = 203, 72.8%). None of these potential covariates met the requirements of having a statistically significant *r* value ≥0.2 and *p* < 0.05 when correlated with the outcome variables and were therefore not included in any of the regression models.

### Prescribing new, novel or different to usual medication

For context and to understand how often participants would typically prescribe new, novel or different to usual medication to patients with newly diagnosed ND, compared to those with advanced stage ND, means and SDs for the outcome measures were generated. These data show that participants reported being less likely to prescribe new, novel or different to usual medication to patients with newly diagnosed ND (mean 51.8%, SD 27.3%), compared to patients with advanced stage ND (mean 60.8%, SD 26.5%). A subsequent paired-samples t-test confirmed that this was a significant difference in outcome between prescribing scenarios (*t* [278]=−4.45, *p* = 0.01).

### Regression results for the long-form 93-item questionnaire

**Fig 1:**
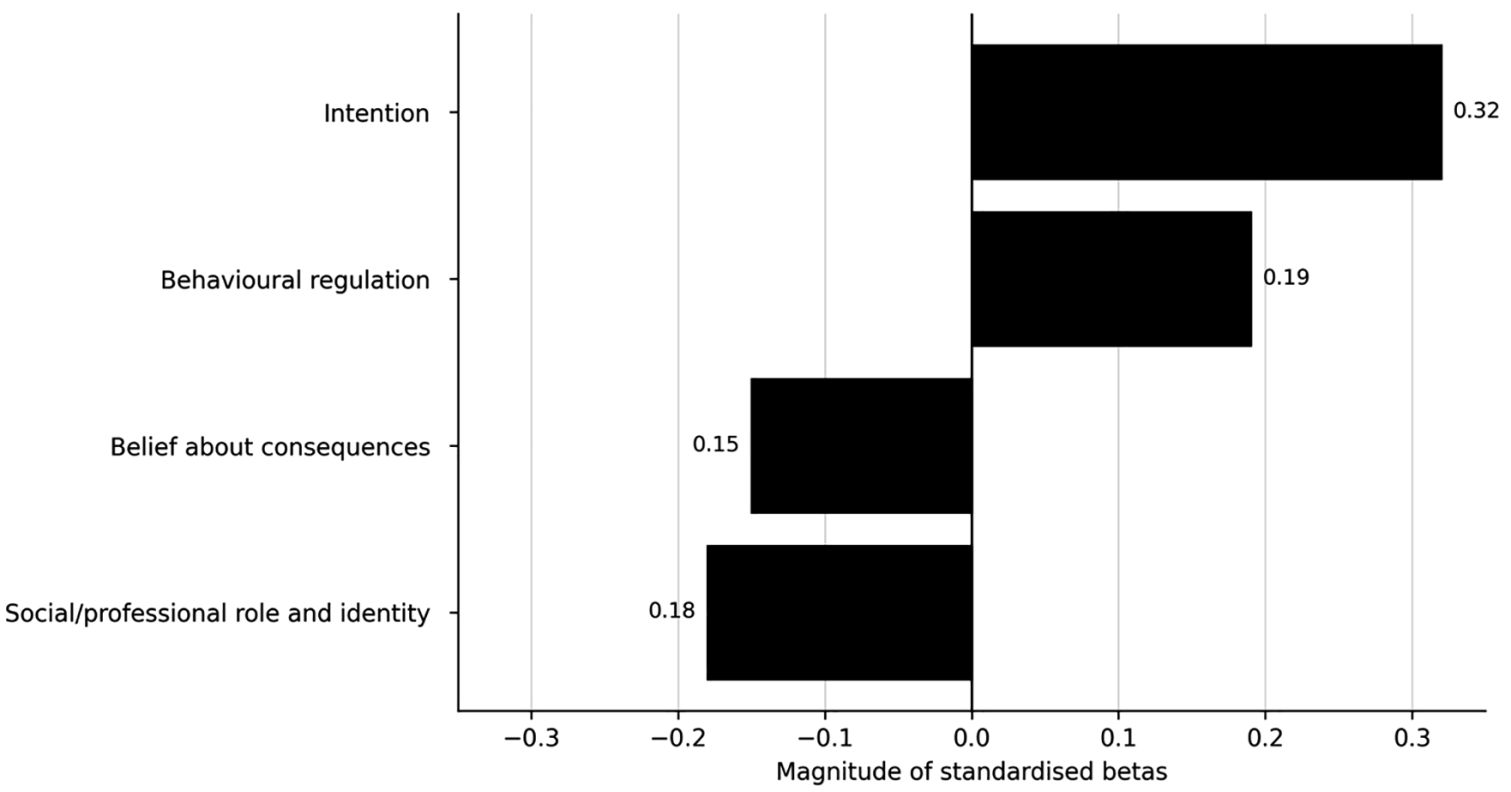
Main predictors of prescribing behaviour for neurodegenerative disease. Horizontal bars show the standardised direction and magnitude of the main predictors identified in the regression models. Positive values indicate predictors associated with greater likelihood of prescribing a new, novel, or different medication, whereas negative values indicate predictors associated with lower likelihood of prescribing.

For scenario 1 (patient with newly diagnosed ND), the stepwise regression generated a statistically significant model comprising three predictors. Intentions and behavioural regulation both positively predicted the likelihood of an HCP prescribing new, novel or different to usual medication (β = 0.32, *p*=<0.001, 95% confidence interval (CI) 4.75, 10.70; β = 0.19, *p* = 0.005, 95% CI 1.57, 8.92). This indicates that HCPs with greater intention (i.e., a conscious readiness to prescribe) and greater behavioural regulation (i.e., an ability to monitor and control one’s prescribing behaviour) are more likely to prescribe new, novel or different to usual medication. Conversely, social/professional role and identity negatively predicted the likelihood of prescribing new, novel or different to usual medication (β = ‑0.184, *p* = 0.002, 95% CI −11.50, −2.54). This indicates that HCPs with greater role responsibility and self-identity (i.e., perceiving prescribing to be an important part of their role and identifying as a prescribing HCP) are less likely to prescribe new, novel or different to usual medication (see Fig 1). Overall, this model explained 18.4% of the variance in how often participants would prescribe new, novel or different to usual medication to a newly diagnosed patient with a ND.

For scenario 2 (patient with advanced stage ND), the stepwise regression model generated a statistically significant model comprising two predictor variables. Intention positively predicted the likelihood of prescribing new, novel or different to usual medication (β = 0.23, *p* < 0.001, 95% CI 2.73, 8.11); thus, as above, HCPs with greater intention are more likely to prescribe new, novel or different to usual medication. Conversely, belief about consequences negatively predicted likelihood of prescribing new, novel or different to usual medication (β = −0.145, *p* = 0.014, 95% CI −6.79, −0.75). This indicates that HCPs who are more concerned about the consequences of prescribing out of habit (i.e., who believe there is more potential risk than benefit) are less likely to prescribe a new, novel or different to usual medication. Overall, this model explained 6.4% of the variance in how often participants would prescribe new, novel or different to usual medication to a patient with advanced stage ND.

Further data from the stepwise regression models are presented in Table 2.

**Table 2:** Regression results for the long-form 93-item questionnaire.

Scenario 1 (newly diagnosed ND)	Intentions	Social/professional role and identity	Behavioural regulation
Unstandardised β	7.72	−7.02	5.25
Standardised β	0.32	−0.18	0.19
*p* value	<0.001	0.002	0.005
***Model statistics***:			
R, R^2^	0.43, 0.18		
R^2^ change	0.02		
Adjusted R^2^	0.16		
F value	20.71		
95% CI	1.57, 8.92		
**Scenario 2 (advanced stage ND)**	**Intentions**	**Belief about consequences**	
Unstandardised β	5.42	−3.77	
Standardised β	0.23	−0.15	
*p* value	<0.001	0.014	
***Model statistics:***			
R, R^2^	0.25, 0.064		
R^2^ change	0.02		
Adjusted R^2^	0.06		
F value	9.50		
95% CI	−6.78, −0.75		

CI, confidence interval; ND, neurodegenerative disease

## Discussion

The purpose of this study was to explore the likelihood of HCPs prescribing a new, novel or different medication for NDs and to map the strongest predictors of prescribing to behaviour change determinants aligned to the TDF and COM-B theoretical frameworks. Overall, when positioned with a hypothetical choice of prescribing a medication different to routine choice, HCPs were more likely to consider prescribing a new, novel or different medication to patients with advanced stage ND (scenario 2) than to patients with newly diagnosed ND (scenario 1). This finding complements the wider literature, which highlights that patient stage of disease is a key factor that influences prescribing choice, [32–34] and that HCPs are more likely to adhere to guideline recommendations on using first-line therapy [35] (i.e., less likely to deviate from routine prescribing for newly diagnosed patients). Ensuring that HCPs are aware of up-to-date guidelines and have access to them is clearly an important gateway to influencing future prescribing changes.

Further, the regression model identified that the behavioural determinants most likely to predict a change in prescribing differ depending on whether HCPs treat newly diagnosed patients or those with advanced stage ND, which supports the proposed hypothesis (see Table 3). Specifically, the data indicated that the behavioural determinant ‘intentions (to prescribe)’ may be a more generic target for changing prescribing behaviour as it was a significant predictor for both new and advance stage patient scenarios, whereas behavioural regulation, social/professional role and identity, and belief about consequences may represent more selective targets relevant in context-dependent scenarios. Collectively, these predictors are hypothetically lucrative targets for an intervention that aims to optimise medication choice in NDs; however, further study is required to test their impact on change.

**Table 3:** Mean values for each of the domains and outcome measures.

Item	Mean value (SD)	Number of observations, n
*Domain*		
Knowledge	5.83 (0.64)	279
Skills	5.28 (0.76)	279
Social/professional role and identity	5.69 (0.64)	279
Belief about capabilities	5.97 (0.63)	279
Optimism	5.78 (0.82)	279
Belief about consequences	3.62 (0.82)	279
Reinforcement	5.11 (0.68)	279
Social influences	5.19 (0.81)	279
Intentions	4.59 (0.84)	279
Goals	5.41 (0.51)	279
Memory, attention and decision processes	4.41 (0.46)	279
Environmental context and resources	4.84 (0.52)	279
Emotions	3.58 (0.76)	279
Behavioural regulation	5.42 (0.99)	279
*Outcome*		
Scenario 1 – likelihood of prescribing	51.83 (27.32)	279
Scenario 2 – likelihood of prescribing	60.83 (26.53)	279

SD, standard deviation

Perceiving the significant predictive factors of this study through a behaviour change lens indicated that HCPs’ psychological capability (led by their behavioural regulation, e.g., how they self-monitor or objectively measure their actions) and reflective motivation (led by their intention, e.g., making conscious decisions) are important positive influences of prescribing. At the same time, other aspects of reflective motivation (led by HCPs’ social/professional role, e.g., how they perceive themselves and their role, and their belief about consequences, e.g., outcome expectancies) may also be important predictors; however, these should be carefully considered as they may negatively predict HCPs’ likelihood of prescribing outside of habit.

In relation to the existing literature, use of behaviour change theory or frameworks to improve prescribing practice is growing. The methods used vary and most target antibiotic or inappropriate prescribing [14,36,37], thus making it difficult to compare and identify causal relationships between the determinants and behaviour change in NDs. That said, behavioural regulation was found to be important (and, therefore, a target behaviour) for improving polypharmacy prescribing in older adults [38] and partly explained a positive relationship in antimicrobial stewardship behaviour [30]. Similarly, behavioural regulation and intention were also identified as important determinants in influencing prescribing nurses to prescribe more medication in community care settings [39]. Conversely, behavioural regulation and intention were not reported by a recent systematic review of interventions designed to optimise prescribing [14]. Rather, the review by Talat *et al*. 2022 [14] identified two interventions that targeted social/professional role and identity, and belief about consequences: determinants this current study also found as negative predictors of prescribing. Nevertheless, there is other evidence that HCP belief about consequences is often an important target of changing prescribing [38–40]. Differences in study findings may be due to the often-missing information about how interventions identify and report prescribing determinants, thus requiring a level of interpretation from the review authors [14]. Although this report found differences in the predictive direction of the determinants identified (two negative and two positive predictors), they still comprise part of COM-B’s reflective motivation, which is theorised to play a key part in habit formation [41], the role of which has been shown to have a positive effect on HCP behaviour [42]. The inconsistencies in the targets of change and their importance highlights the need to test the effectiveness of changing prescribing behaviour for the behavioural determinants reported here.

Understanding prescribing practices in this way fulfils the first stage of the BCW framework [12] and helps to identify relevant theoretically driven intervention content and the circumstances in which there may be a greater likelihood of change [43]. The potential of this approach is highlighted in a recent systematic review, which identified 16 interventions that effectively optimised medication prescribing and, using the TDF (which is compatible with the BCW’s COM-B model), identified relevant behavioural determinants and BCTs that the interventions used to trigger change [14]. Unlike the interventions described in the review by Talat *et al*. 2022 [14], this current study clearly highlights how the behavioural determinants influencing prescribing for NDs were identified. Hence, this work contributes new knowledge specific to the ND field and, by identifying relevant intervention content using the subsequent stages outlined in the BCW framework [12], provides an opportunity to explore the mechanisms of action [44] potentially linked to changing prescribing practice.

This study has several strengths, one of which is that a significantly powered sample of participants was recruited from across Europe, thereby providing a wider perspective on prescribing for NDs. In addition, the data captured enabled the statistical quantification of predictors. Other strengths include the development of a robust survey, which was informed by existing literature, a primary (related) research study, and underpinned by a behaviour change theoretical framework. However, using a newly developed survey poses certain limitations. For example, the survey requires further validation to ensure its robustness. Designing a situational scenario is also another limitation as it does not capture actual prescribing practice and is not sufficient to indicate causality. In addition, the design of the present study ensured that core prescribing competencies were addressed comprehensively and without redundancy across the two situational scenarios. Future research in this area should examine a broader range of prescribing competencies through the inclusion of additional scenarios.

That said, the results are enough to guide the development of future experimental studies to explore the change in prescribing patterns when certain behavioural determinants are targeted. While the aim of the current study was to examine the facilitators of broad behaviour change, future research should investigate whether the findings reported here can also be observed when focusing on specific classes of drugs or even a comparison of different disease states.

Future research should also consider replicating this study longitudinally (for example, pre-/post-launch of a new medication) to explore whether the behavioural determinants identified here are malleable over time and result in prescribing change, a sub group analysis across different countries or job seniority, or if other factors such as sensitivity of missing data also need addressing (see, e.g., [45]).

In conclusion, this study identified a set of generic and more selective behavioural predictors that may be useful targets to help optimise prescribing for NDs in the future. This insight is valuable to those developing behaviour change interventions as it can be used to identify relevant intervention content and offers an opportunity to examine whether there is a causal relationship between the behavioural determinants and outcome changes. The findings of the current study have clear implications for the clinical management of NDs. A better understanding of the factors that influence prescription decisions can help identify more effective pathways to pharmacotherapy. This, in turn, is likely to reduce the healthcare burden and enhance patients’ quality of life.

## Supporting information

S1 File STROBE 2007 (v4) statement and checklist. (PDF)

S2 File Study protocol. (DOCX)
