# Supplementary material for: Theory-based predictors of prescribing behaviour for neurodegenerative diseases: A cross-sectional survey with European healthcare professionals
Source: PLoS One. 2026 Jul 15;21(7):e0353479. doi: 10.1371/journal.pone.0353479 (PMC13372165; doi:10.1371/journal.pone.0353479)
Supplement: S2 File — (DOCX) [file pone.0353479.s002.docx]

- S3. Study protocol

## Background

Deciding which medication is most appropriate for patients is a challenging task for healthcare professionals (HCPs) [1]. HCPs often navigate through a vast, complex and ever-expanding body of medical knowledge to ensure they remain constantly updated with the latest developments on how to treat or manage a disease. Neurodegenerative diseases (NDs), such as Alzheimer’s disease, dementia and Parkinson’s disease, are a leading cause of disability and death worldwide [2]. Further, NDs often have highly variable disease courses and patient responses to medication [3] that may exacerbate the challenge of choosing the best medication. Understanding HCPs’ prescribing practices and factors that influence their choice of medication may be beneficial in optimising the reach of medication for NDs. A wide variety of factors that influence prescribing have already been explored in the literature [4, 5]; however, it is not well understood which factors are most important when determining the best treatment for NDs [6].

It is important to identify and address all the reasons for suboptimal prescribing as any unaddressed reasons can prevent change [6]. However, understanding any behaviour (such as prescribing), and the underlaying drivers that influence it, is complex [7]. Behaviour change theories and frameworks like the Behaviour Change Wheel [8] and Theoretical Domains Framework (TDF) [9] provide a robust and transparent way of managing this complexity and have previously been used to understand and optimise prescribing decisions [6, 10, 11]. Utilisation of such frameworks provides an opportunity to align factors that influence prescribing for NDs to behavioural determinants of change and, upon identifying these determinants, identify theoretical approaches most likely to bring about a change toward more optimal prescribing for NDs.

This research study therefore seeks to understand which behavioural determinants most likely predict HCPs’ likelihood of prescribing a new, novel or different to usual choice of medication for patients who are newly diagnosed or have advanced stage ND. Full ethical approval was granted from Aston University College of Health and Life Sciences Research Ethics Committee.

The protocol for achieving the study aim is outlined below.

## Participants

This study will invite HCPs (such as consultants, nurses, general practitioners or prescribing pharmacists) who meet the following inclusion criteria to take part:

- HCPs who hold a current legal qualification to prescribe medication
- HCPs who have prescribed medication for a patient with a ND in the past 2 years
- HCPs who are currently employed as a HCP
- HCPs who can understand the English language

## Recruitment

This study aims to recruit a minimum of 250 HCPs employed in healthcare across Europe. Prospective participants will be identified using recruitment methods such as, but not limited to, the following:

- Obtain National Health Service (NHS) Health Research Authority ethical approval and recruit HCPs via the NHS Clinical Research Network and sponsored NHS sites
- PubMed PubReMiner ([PubMed PubReMiner: a tool for PubMed query building and literature mining [amc.nl]](https://hgserver2.amc.nl/cgi-bin/miner/miner2.cgi)) is a publicly available database that will be used to identify and email key opinion leaders of published journal articles
- Identify clinics or treatment centres across Europe that treat or manage patients with a ND and email or post study invites
- Recruitment posters will be displayed on neurodegenerative network platforms designated for HCPs
- The same recruitment posters will be displayed at Aston University’s Medical School network. Permission will be sought from the Medical School prior to posting. Where appropriate, we will also seek permission to post and advertise to other Medical Schools across Europe
- Social media, such as Twitter and LinkedIn, will be used to promote the study and encourage participation. The research team will use their personal or professional accounts to share the recruitment advertisement
- We will use snowballing to identify other HCPs among those who already expressed interest in taking part

## Participant consent

Participants will be invited to provide consent anonymously online by checking boxes to indicate they have read the participant information sheet and are happy to continue with taking part.

## Development of the survey

- The content of an online survey will be designed using findings from a rapid review of the literature and focus group with HCPs from an aligned previous study
- The survey will be designed using the 14 behaviour determinants aligned to the TDF as a structure for grouping questions
- The platform Qualtrics will be used to develop and host the survey

Table 1. Example of content from the long-form 93-item questionnaire: A full copy of the questionnaire has not been published because the contents are commercially sensitive

| TDF domain | Number of items | Example item |
| --- | --- | --- |
| Knowledge | 8 | I am aware of the local/national guidelines for prescribing the recommended medication |
| Skills | 6 | I require more training and experience to improve my prescribing practice |
| Social/professional role and identity | 4 | It is my job as a practicing clinician to only prescribe medication that I am familiar with |
| Belief about capabilities | 4 | My past experiences of prescribing have prepared me well for choosing which medication to prescribe in the future |
| Optimism | 2 | Regarding the medication I prescribe, I usually expect the best outcome(s) |
| Belief about consequences | 9 | I believe my patients who are prescribed more than one medication are more likely to be worse off if I prescribe a new, different or substitute medication |
| Reinforcement | 6 | If I discover a new or different medication that I like, I usually continue prescribing it for a while |
| Intentions | 3 | During my next shift, I intend to consider prescribing a new or different medication |
| Goals | 7 | For me, the primary goal in my role as a clinician is to prescribe medication that I know patients will adhere to |
| Memory, attention and decision processes | 23 | I like to leave risky decisions to others (10 items reflect those of a validated measure on defensive decision making [12]) |
| Environmental context and resources | 8 | During consultations there is enough time to consider prescribing a new or different medication |
| Social influences | 8 | I value my colleagues’ opinions |
| Emotions | 10 | When I prescribe a medication that is different to my usual choice, I feel agitated |
| Behavioural regulation | 3 | Prescribing a new or different medication following the guidelines is something I do automatically |

TDF, Theoretical Domains Framework

## Short-form 14-item visual analogue scale

A short-form 14-item questionnaire was also developed to assess the potential of capturing the same outcome data using a more parsimonious version of the survey and a more sensitive response scale. As such, visual analogue scale (VAS) items were created by using one item that represented each of the 14 domains of the long-form 93-item questionnaire. Participants were invited to indicate, ‘to what extent do the following factors influence the choice of medication you prescribe?’ for each VAS item, with a response scale ranging from 0=never to 100=always. This was presented after completion of the main 93-item survey. These data are not presented or analysed here.

## Survey procedure

- All prospective participants will be sent a study email invite containing study information and a URL link to the online survey. They will also be given the option to opt out of any follow-up emails
- Participants who chose to take part will do so by clicking on the URL link at their own discretion
- Upon clicking the URL link, participants will be presented with a welcome message from the study investigator and encouraged to click on the arrow indicating the next page
- Participants will first be prompted to complete eligibility questions. Those who fail to meet the study requirements will not be able to continue and thanked for their time and interest
- Participations will then be asked to read the participant information sheet and provided with the contacts detail of the study investigator should they have any questions
- Following this, participants will be asked to complete their informed consent to take part
- Once consent is collected, the survey will begin. It is estimated to take participants approximately 25 minutes (including reading the participant information sheet) to take part
- First, participants will be asked to answer a series of demographic questions
- Secondly, participants will be presented with two scenarios and asked to rate (from 0 = never to 100 = always) their likelihood of prescribing a new, novel or different to usual medication for patients who have been newly diagnosed or with advanced stage ND. These scenarios will be the dependent variable outputs that the study measures
- Thirdly, participants will be prompted to answer 14 sets of questions (ranging from 3 to 23 questions in each set) based on their experience of prescribing medication to patients with a ND
- Once all questions have been answered, participants will be presented with a screen thanking them for their time. Participants will also be notified of an opportunity to enter a charity prize draw, whereby a donation will be made to a ND charity of their choice. If interested, participants will be prompted to complete their details on a separate online form (also hosted by Qualtrics) to enable to draw to take place. Any participant details collected will be kept and stored separately to any survey responses. The draw will comprise one £100 donation and two £50 donations

## Data analysis

Data collected from the online survey will be downloaded from Qualtrics onto Aston’s server ‘Box’. Once downloaded, it will be processed in Microsoft Excel and imported in SPSS where it will be quantitatively analysed using descriptive and logistic regression analysis. The researchers will use Aston’s Virtual Private Network to access the dataset. Only Aston researchers who lead on this project and have a username and password will have access to these files.

## Data availability

The research project is part of a larger knowledge transfer initiative funded by the UK government, designed to foster collaboration between academia and industry to break down traditional barriers. It is part funded by Innovate UK and industry partner Alpharmaxim Limited. Such initiatives operate under a structured framework to ensure equitable contributions from both academic and industry partners. Alpharmaxim’s involvement was instrumental in refining the research focus, particularly in identifying the specificity of the research question and maintaining the project’s alignment with its intended objectives. Alpharmaxim’s expertise and practical insights significantly enriched the study and ensured that our research remained targeted and impactful throughout its development.

Alpharmaxim identified a unique opportunity to revolutionise healthcare communications campaigns, using innovative application of behavioural science research. This is important because pharmaceutical companies’ marketing campaigns for drugs have followed the same model for years (using the same templates out of habit) and there are currently limited means to measure their success. Alpharmaxim is passionate about driving change in how healthcare communications campaigns are developed, by focusing on identifying beliefs and barriers that lead to behavioural changes, enabling much-needed drugs to reach patients. As is the nature with all work carried out within a Knowledge Transfer Partnership project, the industry partner will have full access to the completely anonymised data set.

## References

1. Cairo Notari S, Sader J, Caire Fon N*, et al.* Understanding GPs' clinical reasoning processes involved in managing patients suffering from multimorbidity: a systematic review of qualitative and quantitative research. *Int J Clin Pract* 2021;75:e14187. doi: 10.1111/ijcp.14187

2. Deuschl G, Beghi E, Fazekas F*, et al.* The burden of neurological diseases in Europe: an analysis for the Global Burden of Disease Study 2017. *Lancet Public Health* 2020;5:e551–e67. doi: 10.1016/S2468-2667(20)30190-0

3. Bloem BR, Okun MS, Klein C. Parkinson's disease. *Lancet* 2021;397:2284–303. doi: 10.1016/S0140-6736(21)00218-X

4. Orayj K, Lane E. Patterns and determinants of prescribing for Parkinson’s disease: a systematic literature review. *Parkinsons Dis* 2019;2019:9237181. doi: 10.1155/2019/9237181

5. Wang SY, Groene O. The effectiveness of behavioral economics-informed interventions on physician behavioral change: a systematic literature review. *PLoS One* 2020;15:e0234149. doi: 10.1371/journal.pone.0234149

6. Talat U, Schmidtke KA, Khanal S*, et al.* A systematic review of nudge interventions to optimize medication prescribing. *Front Pharmacol* 2022;13:798916. doi: 10.3389/fphar.2022.798916

7. Michie S, van Stralen MM, West R. The behaviour change wheel: a new method for characterising and designing behaviour change interventions. *Implementation Science* 2011;6:1–12. doi: 10.1186/1748-5908-6-42

8. Michie S, Atkins L, West R. The Behaviour Change Wheel: A Guide to Designing Interventions. London: Silverback Publishing 2014.

9. Cane J, O’Connor D, Michie S. Validation of the theoretical domains framework for use in behaviour change and implementation research. *Implement Sci* 2012;7:37. doi: 10.1186/1748-5908-7-37

10. Ju I, Park T, Ohs JE. Consumer engagement with prescription medicine decisions: influences of health beliefs and health communication sources. *Health Commun* 2020;35:135–47. doi: 10.1080/10410236.2018.1545336

11. Chater AM, Williams J, Courtenay M. The prescribing needs of community practitioner nurse prescribers: a qualitative investigation using the theoretical domains framework and COM-B. *J Adv Nurs* 2019;75:2952–68. doi: 10.1111/jan.14170

12. Marx‐Fleck S, Junker NM, Artinger F*, et al.* Defensive decision making: operationalization and the relevance of psychological safety and job insecurity from a conservation of resources perspective. *J Occup Organ Psychol* 2021;94:616–44. doi: 10.1111/joop.12353
